# Idealized vs. Realistic Microstructures: An Atomistic Simulation Case Study on *γ*/*γ*^′^ Microstructures

**DOI:** 10.3390/ma10010088

**Published:** 2017-01-23

**Authors:** Aruna Prakash, Erik Bitzek

**Affiliations:** Materials Science and Engineering, Institute I (MSE I), Friedrich-Alexander-Universität Erlangen-Nürnberg (FAU), Martensstrasse 5, Erlangen 91058, Germany; erik.bitzek@fau.de

**Keywords:** Ni-base superalloys, *γ*/*γ*^′^ microstructure, atomistic simulations, misfit stresses, experimentally-informed microstructures, virial atomic stresses, finite element simulations, periodic boundary conditions, thermal misfit

## Abstract

Single-crystal Ni-base superalloys, consisting of a two-phase *γ*/$\gamma^{\prime }$ microstructure, retain high strengths at elevated temperatures and are key materials for high temperature applications, like, e.g., turbine blades of aircraft engines. The lattice misfit between the *γ* and $\gamma^{\prime }$ phases results in internal stresses, which significantly influence the deformation and creep behavior of the material. Large-scale atomistic simulations that are often used to enhance our understanding of the deformation mechanisms in such materials must accurately account for such misfit stresses. In this work, we compare the internal stresses in both idealized and experimentally-informed, i.e., more realistic, *γ*/$\gamma^{\prime }$ microstructures. The idealized samples are generated by assuming, as is frequently done, a periodic arrangement of cube-shaped $\gamma^{\prime }$ particles with planar *γ*/$\gamma^{\prime }$ interfaces. The experimentally-informed samples are generated from two different sources to produce three different samples—the scanning electron microscopy micrograph-informed quasi-2D atomistic sample and atom probe tomography-informed stoichiometric and non-stoichiometric atomistic samples. Additionally, we compare the stress state of an idealized embedded cube microstructure with finite element simulations incorporating 3D periodic boundary conditions. Subsequently, we study the influence of the resulting stress state on the evolution of dislocation loops in the different samples. The results show that the stresses in the atomistic and finite element simulations are almost identical. Furthermore, quasi-2D boundary conditions lead to a significantly different stress state and, consequently, different evolution of the dislocation loop, when compared to samples with fully 3D boundary conditions.

## 1. Introduction

Ni-base superalloys are an excellent class of high-temperature materials that are used as single-crystal turbine blades in aircraft engines and power plants [1]. Comprised of a two-phase *γ*/$\gamma^{\prime }$ microstructure, these single crystals exhibit the ability to withstand high thermo-mechanical loads at temperatures well above 1200 K. The ordered $\gamma^{\prime }$-phase in a L1${}_{2}$ crystal structure (comprised primarily of Ni${}_{3}$Al) occupies a volume fraction between approximately 40% and 70%, thereby geometrically constraining the plastic deformation in the disordered *γ* matrix comprised primarily of Ni in the fcc structure [2]. The high strength of these two-phase alloys is a direct result of the coherent interface formed between the *γ*/$\gamma^{\prime }$ phases [3].

The lattice misfit between the *γ* and $\gamma^{\prime }$ phases results in internal stresses in the microstructure. These misfit stresses have a significant influence on the mechanical and high temperature creep behavior of the material. For instance, misfit stresses are known to influence the evolution of the shape of the precipitates at elevated temperatures [4]. The stresses may be relieved by the formation of an interface dislocation network during initial stages of low stress high temperature creep, a process usually accompanied by a change in the precipitate shape (rafting) [5,6,7]. It is hence evident that the incorporation of the appropriate misfit stress state is paramount towards modeling the mechanical behavior of *γ*/$\gamma^{\prime }$ microstructures.

Atomistic simulations, especially of the molecular dynamics/statics kind, have now become a cornerstone of computational materials science. The atomic resolution offered by such simulations techniques has led to unprecedented insights into the deformation behavior and failure of materials [8,9]. Notwithstanding the importance of the interatomic potential that defines the interaction between atoms, the accuracy and predictive capability of such simulations depend primarily on two factors, viz. the ability to incorporate realistic initial structures, preferably a one-on-one reproduction of experimental microstructures, and the exactitude of applied boundary conditions with real-world conditions.

To date, most atomistic simulations of *γ*/$\gamma^{\prime }$ microstructures are, however, performed with rather simplistic setups and boundary conditions [10,11,12,13,14,15,16]. Such simplistic simulation setups and boundary conditions are indeed required for quantitative studies. However, they can easily mask or inhibit certain mechanisms that are quintessential in determining the deformation characteristics of the material; see e.g., [17]. Studies of a qualitative nature that focus on deformation mechanisms should hence use such simplistic structures with extreme caution.

Although a complete atom-by-atom reconstruction of structures is not yet possible due to the lack of atomic resolution in many experimental methods, realistic features, like curvature, topology, etc., can be accounted for by using experimental data in simulations. With the availability of advanced sample generation techniques and tools [18,19], there is now an impetus towards the inclusion of more realistic microstructures in simulations, and this has led to the new class of experimentally-informed atomistic simulations [17,20]. This synergy between experiments and simulations is indeed necessary to validate and verify multiscale modeling techniques, which have become almost incomparable in the level of detail and the amount of data generated [21]. Robust and reliable multiscale tools that are driven by such synergy can open new research avenues in materials science and mechanics, like the theory-guided design of material structures with desired properties that are defined a priori [22].

Nonetheless, the mere usage of experimentally-informed samples in atomistic simulations may not be sufficient enough. Such models often necessitate the usage of boundary and loading conditions that might be more restrictive than real-world scenarios, and consequently, change the internal stress state, and thus, the resulting properties of the material. To the best of our knowledge, no study has been performed on the usage of realistic samples and accompanying boundary conditions on the stress state in atomistic simulations.

In this work, we compare typical idealized simulation geometries with samples derived from experimentally-determined morphologies and the accompanying boundary conditions, with respect to the misfit stress state in model *γ*/$\gamma^{\prime }$ microstructures, using atomistic simulations. The idealized samples are motivated from a periodic arrangement of perfect cuboid-shaped $\gamma^{\prime }$ particles embedded in a *γ* matrix. To obtain realistic structures, we generate two atomistic samples using an atom probe tomography dataset and a further sample by digitizing a scanning electron microscope (SEM) micrograph. The misfit stress state in the commonly-used $\gamma^{\prime }$-cube-in-*γ*-matrix atomistic sample is, furthermore, compared to a finite element simulation to evaluate the atomic stress state. Subsequently, we study the influence of the resulting misfit stress state on the evolution of a dislocation loop under tensile loading.

We note here that throughout the manuscript, the terms experimentally-informed and realistic are used interchangeably, to describe samples that account for realistic features like curvature, topology, etc. It must, however, be stressed that although the experimentally-informed samples are more realistic in terms of geometry and morphology, and may have a more adapted composition, they are far removed from real microstructures, which have different lattice constants, more complex composition and probably contain additional defects. The rest of the paper is organized as follows: In Section 2, we provide the details on the methods used, including particulars on the generation of atomistic and finite element samples. The results, viz. the misfit stresses and the evolution of a dislocation loop, are presented in Section 3. Subsequently, the results are discussed in Section 4, and concluding remarks are presented in Section 5.

## 2. Methods

### 2.1. Sample Geometry

The samples used in the current work are classified, based on the origin of the topological features forming the *γ*/$\gamma^{\prime }$ microstructure, as idealized samples and realistic (experimentally-informed) samples.

#### 2.1.1. Idealized Samples

**Figure 1:**
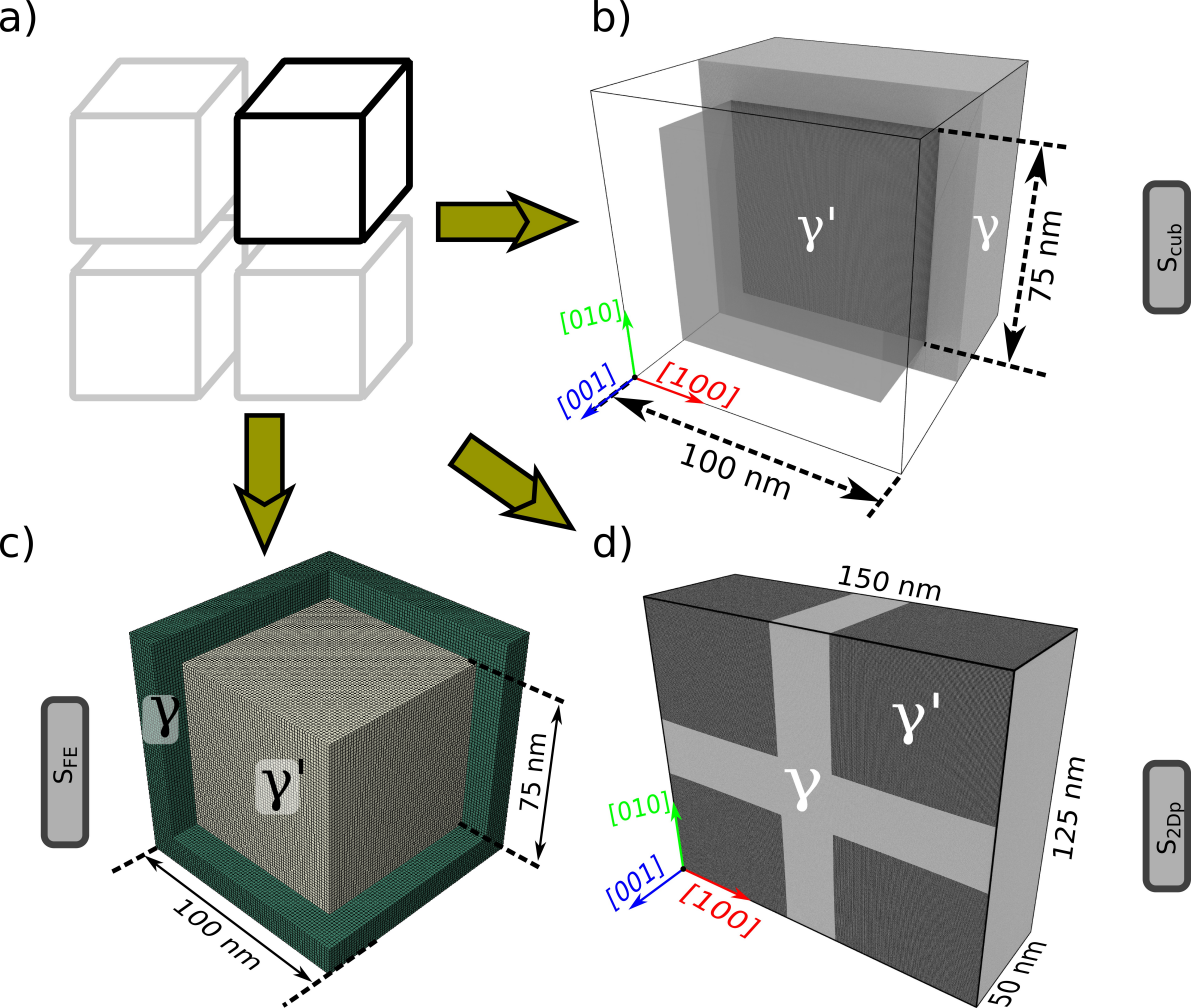
Simulation setup of the idealized samples used in the current work. (**a**) Schematic picture of the idealized *γ*/$\gamma^{\prime }$ microstructure showing cube-shaped $\gamma^{\prime }$ particles embedded in a matrix; (**b**) Atomistic model of the idealized *γ*/$\gamma^{\prime }$ microstructure. Due to symmetry, only one cube-shaped $\gamma^{\prime }$ particle, highlighted in black in (a), of a length of 75 nm embedded in a matrix of a width of 25 nm, is used in the simulations. The atomistic sample contains approximately 90 million atoms. periodic boundary conditions (PBCs) are imposed in all directions. The $\gamma^{\prime }$ particle is shown as a transparent surface for visualization purposes; (**c**) Meshed structure for FE simulations. The dimensions correspond to that of the atomistic structure in (a). PBCs are imposed in all directions using external control nodes. The elements in $\gamma^{\prime }$ are colored grey, whilst those in *γ* are colored green; (**d**) Quasi-2D simulation sample with planar interfaces. This setup corresponds to a planar cut of the idealized *γ*/$\gamma^{\prime }$ microstructure and extrusion along the thickness direction so as to obtain two orthogonal and two parallel channels. PBCs are imposed only along the thickness (z||[001]) direction. Color code (for the atomistic simulation samples): Ni atoms are colored grey, whilst the Al atoms are colored black.

The idealized samples (see Figure 1) are motivated from the periodic arrangement of perfect cube-shaped $\gamma^{\prime }$ particles embedded in a *γ* matrix as shown in Figure 1a. Such sample setups, or slightly modified versions thereof, are regularly used in atomistic (e.g., [23,24]), mesoscale (e.g., [25,26]) and continuum scale (e.g., [27,28]) simulations. The first atomistic sample, *S*${}_{cub}$, replicates a perfect $\gamma^{\prime }$ cube of size 75 × 75 × 75 nm${}^{3}$, surrounded by a *γ* channel of width 25 nm. The dimensions of the simulation box are 100 × 100 × 100 nm${}^{3}$, with periodic boundary conditions (PBCs) imposed along all three directions (see Figure 1b).

A corresponding finite element (FE) sample (Figure 1c), *S*${}_{FE}$, whose dimensions are identical to that of sample *S*${}_{cub}$, is also used in the current work. To ensure congruence with the boundary conditions of sample *S*${}_{cub}$, and to enable a direct comparison of the resulting misfit stress state with that of sample *S*${}_{cub}$, PBCs are also imposed on the FE mesh. This is accomplished by using fictitious constraints through which the displacement of two equivalent points *a* and *b* located on opposite surfaces of the cube-shaped simulation box are coupled with the macroscopic deformation gradient (${\bar{F}}_{ij}$) as follows [29]:

(1) $$u_{i}^{a}-u_{i}^{b}=a_{j}{\bar{F}}_{ij},\;i,j=1\ldots 3$$

The components of the macroscopic deformation gradient can be imposed through the displacement of so-called control nodes, which are additional/auxiliary nodes introduced in the three principal Cartesian directions:

(2) $$u_{i}^{p}={\bar{F}}_{ip},\;i,p=1\ldots 3,$$

where the superscript *p* refers to the index of the auxiliary node under consideration. It can be shown, through the discretized weak form of the boundary value problem, that the reaction forces on the auxiliary nodes weighted by the volume of the unit cell correspond to the components of the macroscopic first Piola–Kirchhoff (PK) stress (${\bar{P}}_{ij}$) tensor [30]. Consequently, we can prescribe, component-wise, either the deformation gradient or the first PK stress tensor.

The second idealized atomistic sample, *S*${}_{2Dp}$, represents a planar cut (with a thickness of 50 nm) along a plane containing four $\gamma^{\prime }$ particles. The sample hence reflects a quasi-2D structure that contains four channels orthogonal to each other, as shown in Figure 1d. PBCs are imposed only along the thickness direction (i.e., z||[001]); atoms inside a boundary layer of 2 nm are fixed in the other two directions.

#### 2.1.2. Realistic Samples

**Figure 2:**
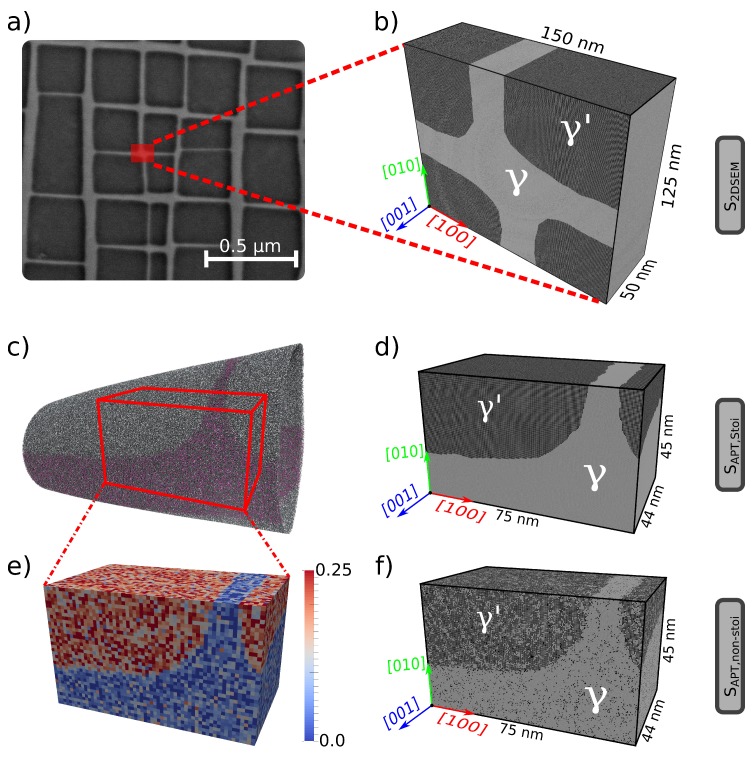
Simulation setup of the realistic samples used in the current work. (**a**) SEM-micrograph of a Ni-base superalloy Astra1 with the *γ*/$\gamma^{\prime }$ microstructure [31]. The atomistic sample corresponds to the region of interest marked in red; (**b**) SEM micrograph-informed quasi-2D atomistic sample with dimensions identical to sample S2p in Figure 1d. The channel thickness is 25 nm, and PBCs are imposed only along the thickness of the sample; (**c**) Atom probe tomography (APT) dataset with only Re (magenta), Ni (grey) and Al (black) atoms shown. Only the cuboidal region marked in red is used for further sample generation; (**d**) APT-informed stoichiometric atomistic sample; (**e**) Local concentration of Al in the region of interest in the APT sample; (**f**) APT-informed non-stoichiometric atomistic sample using the local concentrations of Al and Ni in the original APT data. Color code (for the atomistic simulation samples): Ni atoms are colored grey, whilst the Al atoms are colored black.

The second set of samples, i.e., realistic samples (Figure 2), are generated directly from experimental data. Two different experimental datasets are used for the generation of atomistic samples in the current work—a scanning electron microscopy (SEM) micrograph and atom probe tomography data.

The first realistic sample, *S*${}_{2DSEM}$, is constructed by digitizing a micrograph obtained from SEM investigations [31]. A region of interest (marked in red in Figure 2a), consisting of four $\gamma^{\prime }$ particles, is extracted out of the complete micrograph. This region of interest is chosen so as to have a balance between reasonable computation times and the incorporation of topologically-relevant features in the experimental dataset. By using iso-surfaces of the grayscale in the micrograph, we first identify the inter-phase boundary (IPB) and generate the sample geometry by constructing a surface mesh around either the *γ* matrix or the $\gamma^{\prime }$ particles. A detailed description of the steps involved in generating such SEM micrograph informed samples is provided in [32]. The simulation box has the dimensions of 150 × 125 × 50 nm${}^{3}$. Due to its quasi-2D nature, PBCs can only be imposed along the thickness of the sample. As with sample *S*${}_{2Dp}$, atoms in a boundary layer of a thickness of 2 nm are fixed in the other two directions.

The second realistic sample, *S*${}_{APT,stoi}$, is an atom probe tomography (APT)-informed atomistic sample, similar to the sample used in [17]. APT measurements provide information on the position and chemical species of individual atoms in a needle-shaped specimen and can hence be directly used in conjunction with atomistic simulations. Using iso-density surfaces of any ion that partitions strongly into either *γ* or $\gamma^{\prime }$, we identify the shape of the precipitate and construct the specimen geometry. Details on the generation of APT-informed atomistic samples are provided in [17,32]. The so-generated simulation box has dimensions of 75 × 45 × 44 nm${}^{3}$.

The availability of the information of chemical species in the original APT dataset allows us to generate samples with locally-varying chemical composition. Using a voxelized approach, such changes in the atoms of interest (here, Ni and Al) can directly be incorporated in the atomistic samples. This is done by first generating a stoichiometric sample, i.e., sample *S*${}_{APT,stoi}$, and stochastically replacing Ni and Al atoms, such that the resulting local concentration conforms to that of the voxelized data. The resulting structure is hence an APT-informed non-stoichiometric atomistic sample, similar to the sample used in [17] and denoted here as sample *S*${}_{APT,non-stoi}$. In both APT-informed samples *S*${}_{APT,stoi}$ and *S*${}_{APT,non-stoi}$, 3D fixed boundary conditions are imposed along a boundary layer of 2 nm in all three directions.

All aforementioned atomistic samples are generated using the open-source program *nanoSCULPT* [33] with a characteristic orientation of the [1 0 0] ||x-axis and the [0 0 1] ||z-axis. The same orientation is also used in the FE sample *S*${}_{FE}$ for defining the elastic constants of the *γ* and $\gamma^{\prime }$ phases. In general, the orientation of the specimen, particularly in the case of experimentally-informed samples, is obtained from complementary experiments, e.g., electron back-scatter diffraction or X-ray diffraction. For further information on the details of the generation of atomistic samples used in the current work, particularly the experimentally-informed samples, the reader is referred to [32].

Table 1 summarizes the similarities and differences between the samples. Samples *S*${}_{cub}$ and *S*${}_{FE}$ are identical in specimen geometry and boundary conditions (BCs) and, hence, allow for a direct comparison of the misfit stress state. Specimens *S*${}_{2Dp}$ and *S*${}_{2DSEM}$ have identical simulation-box sizes and are subjected to identical BCs, but differ in the way interfaces are modeled (planar vs. realistic curvature). Samples *S*${}_{APT,stoi}$ and *S*${}_{APT,non-stoi}$ allow us to study the influence of chemical composition. Furthermore, whilst the atomistic samples *S*${}_{cub}$, *S*${}_{APT,stoi}$ and *S*${}_{APT,non-stoi}$ incorporate 3D BCs, samples *S*${}_{2Dp}$ and *S*${}_{2DSEM}$ are restricted to quasi-2D BCs, which is a direct result of their construction procedure and specimen geometry. This wide array of samples allows us to clearly delineate the influence of BCs in atomistic simulations.

### 2.2. Simulation Details

All atomistic simulations in the current work are performed using the embedded atom method (EAM) potential of Mishin for Ni and Al [34], which has been shown to represent well the equilibrium properties and defects of both the *γ* and $\gamma^{\prime }$ phases. We note that the two phases, *γ* and $\gamma^{\prime }$, are modeled here as Ni in fcc and Ni${}_{3}$Al in L1${}_{2}$ crystalline structures, respectively. The potential defines the lattice constants of Ni and Ni${}_{3}$Al as 3.52 and 3.57 Å, respectively, resulting in a positive misfit of δ=1.45% between the *γ* (Ni) and $\gamma^{\prime }$ (Ni${}_{3}$Al) phases. The average lattice constant of 3.545 Å is used for both the *γ* and $\gamma^{\prime }$ phases to ensure a coherent interface and thereby eliminating a misfit dislocation network. As mentioned before, both *S*${}_{APT,stoi}$ and *S*${}_{APT,non-stoi}$ are created identically using the same lattice constant; atoms are then replaced stochastically in *S*${}_{APT,non-stoi}$, so that the local atomic concentration corresponds to that found in the original APT dataset. This stochastic replacement can result in a slightly different lattice constant than that found in the stoichiometric samples.

Atoms in an outer layer of 2 nm are fixed in the *x* and *y* directions in samples *S*${}_{2Dp}$ and *S*${}_{2DSEM}$ and in the *x*, *y* and *z* directions in samples *S*${}_{APT,stoi}$ and *S*${}_{APT,non-stoi}$. No atoms need to be fixed in sample *S*${}_{cub}$, since PBCs are imposed in all three directions. All samples are then relaxed using the Fast Inertial Relaxation Engine (FIRE) algorithm [35], whilst simultaneously ensuring zero pressure in all three directions. The resulting stress state is hence a direct consequence of the misfit between the different phases.

A single dislocation loop is then inserted in the relaxed configuration of the atomistic samples following the procedure detailed in [36]. The Burgers vector of the inserted dislocation loop is $a_{0}/2\left[\bar{1}10\right]$ on the (1 1 1)-plane (b=AB(d) in the Thomson tetrahedron convention). In samples *S*${}_{cub}$, *S*${}_{2Dp}$ and *S*${}_{2DSEM}$, we introduce a dislocation loop of radius 15 nm, while due to the reduced simulation box sizes in samples *S*${}_{APT,stoi}$ and *S*${}_{APT,non-stoi}$, we use a smaller loop of radius 7.5 nm. The sample is then relaxed using the micro convergence algorithm [37] to remove local disturbances and to obtain a realistic core structure of the dislocation loop.

Such a dislocation loop is, however, unstable and requires a resolved shear stress (RSS) to avoid its collapse. A relation for this equilibrium RSS $\tau_{c}$ is provided by Scattergood and Bacon [38]:

(3) $$\tau_{c}=\frac{\mu^{\prime }b}{2\pi 2R}\left(ln\frac{2R}{r_{0}}+1.56\right),$$

where *R* is the radius of the loop, $\mu^{\prime }$ is the effective shear modulus, *b* is the magnitude of the Burgers vector and $r_{0}\approx b$ is the radius of the dislocation core. Using the shear modulus of $\mu^{\prime }=124$ GPa as defined by the potential, we obtain an RSS of 1.07 and 1.91 GPa for loops of radii 15 and 7.5 nm, respectively. For uniaxial tension in the *x*-direction, we obtain a Schmid factor of 0.408 for the slip system under consideration and an elastic modulus of 125 GPa, resulting in effective strains of approximately 2.1% and 3.8%, respectively, which are then applied to the simulation box under consideration by homogeneously scaling the atoms in a uniaxial direction; the directions orthogonal to the straining direction are kept stress free. Molecular dynamics simulations at constant strain and 0 K are then performed using the NVE ensemble to track the evolution of the dislocation loop.

For the finite element simulation, anisotropic elastic constants—$c_{11}$ = 246.5 GPa, $c_{12}$ = 147.3 GPa, $c_{44}$ = 124.7 GPa for *γ* and $c_{11}$ = 236 GPa, $c_{12}$ = 154 GPa, $c_{44}$ = 127 GPa for $\gamma^{\prime }$—as defined by the interatomic potential [34] were used. The simulation procedure involves determining the thermal misfit and follows that adopted generally in the literature [39,40], albeit now with PBCs along all directions. The thermal expansion coefficients were chosen so as to introduce effectively the same overall misfit of δ=1.45% as that in the atomistic samples.

## 3. Results

### 3.1. Misfit Stresses

**Figure 3:**
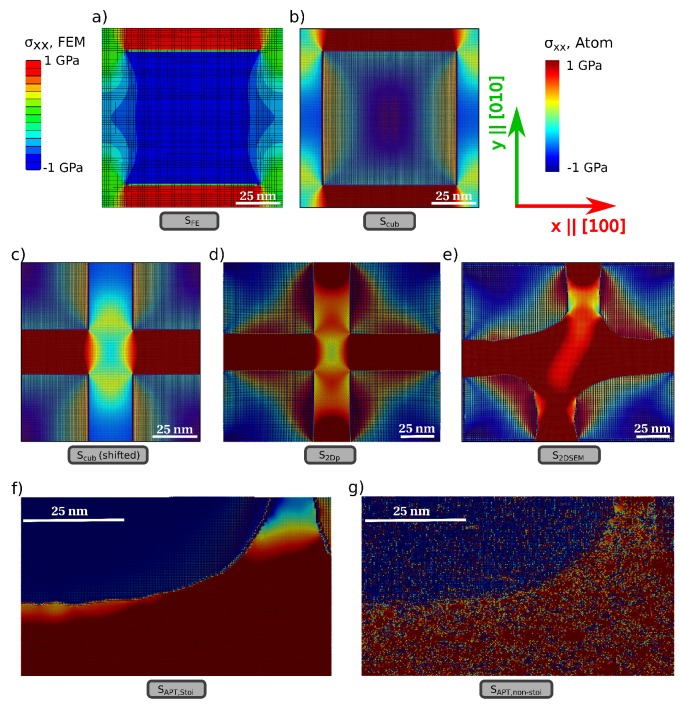
Internal stress distribution in the different samples used in the current work: (**a**) FEM sample *S*${}_{FE}$; (**b**) Atomistic sample *S*${}_{cub}$; (**c**) To help facilitate comparison with other atomistic samples, sample *S*${}_{cub}$ is shifted periodically so that the channels are now in the center of the picture; (**d**) Atomistic sample *S*${}_{2Dp}$; (**e**) Atomistic sample *S*${}_{2DSEM}$ obtained by digitizing SEM micrograph; (**f**) APT-informed atomistic sample *S*${}_{APT,stoi}$ with stoichiometric chemical composition; (**g**) APT-informed atomistic sample *S*${}_{APT,non-stoi}$ with non-stoichiometric chemical composition. All samples are oriented such that x∥[100] and y∥[010]. All atomistic samples share the same color bar.

Due to the differences in the lattice parameters and elastic constants of the *γ* (here, Ni) and $\gamma^{\prime }$ (here, Ni${}_{3}$Al) phases as defined by the potential, relaxation of the atomistic structures results in misfit stresses in the structure. Figure 3 shows the internal stress distribution in the different samples obtained as a result of the lattice misfit; the stress component ($\sigma_{xx}$) shown corresponds to the direction of straining for the dislocation loop introduced subsequently. The stresses in the atomistic case are computed using the virial expression [41], with the corresponding atomic volume determined by a Voronoi construction in the open-source visualization tool OVITO [42].

**Figure 4:**
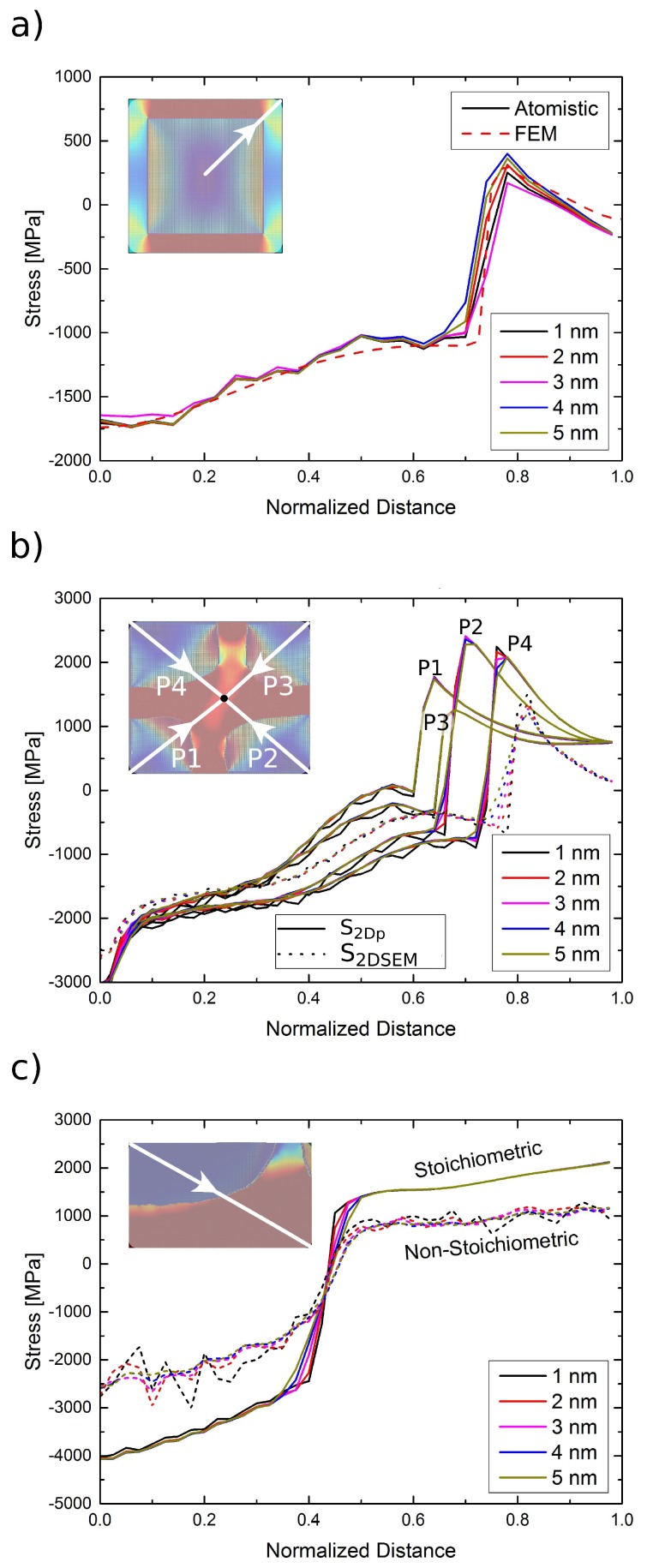
Stress profile (stress component $\sigma_{xx}$) along an internal face diagonal in the different samples. The path used in each sample is marked in white in the corresponding sample (see inset). (**a**) Atomistic sample *S*${}_{cub}$ and FEM sample *S*${}_{FE}$; (**b**) Atomistic samples *S*${}_{2Dp}$ and *S*${}_{2DSEM}$; four different paths are used in sample *S*${}_{2DSEM}$. Since the corresponding paths are identical in sample *S*${}_{2Dp}$, only one path is shown. (**c**) APT-informed stoichiometric sample *S*${}_{APT,stoi}$ and non-stoichiometric sample *S*${}_{APT,non-stoi}$. For all atomistic samples, stress profiles were obtained by extracting atoms inside a cylinder along the path considered. Results for five different cylinder radii (r=1,2,3,4,5 nm) are shown.

The larger lattice constant of the $\gamma^{\prime }$ phase results essentially in a positive misfit structure with tensile stresses in the *γ* channels. The stress distribution in the atomistic sample *S*${}_{cub}$ is both qualitatively and quantitatively similar to that of the FE computation, as seen in Figure 4, although the simulation procedures and the method of generating these misfit stresses is completely different in the atomistic and FE cases. Some minor differences exist: the atomic stresses of Ni atoms in $\gamma^{\prime }$ vary significantly from atom to atom. This is mainly due to the different possible bonding environments of Ni in Ni${}_{3}$Al, resulting in apparent visual differences in the stresses in $\gamma^{\prime }$, for instance, close to the interfaces in the x||[100] direction. Such differences can be eliminated by averaging these atomic stresses over a certain volume. A more detailed discussion of such differences in atomic stresses is presented in Section 4.

Stress states across the atomistic samples, however, vary distinctly from one another. In sample *S*${}_{cub}$, the stress component $\sigma_{xx}$ is significantly different in the orthogonal and parallel channels. By contrast, in the SEM image-informed atomistic sample *S*${}_{2DSEM}$, an almost identical stress state is observed in both the orthogonal and parallel channels. Comparable trends as sample *S*${}_{cub}$ can be observed in the APT-informed atomistic sample *S*${}_{APT,stoi}$; here, as a consequence of the curved interface, a much smoother transition of stresses between the orthogonal and vertical channels is seen. Furthermore, as a consequence of the difference in local chemistry, a substantially heterogeneous distribution of the atomic stresses is observed in the non-stoichiometric sample *S*${}_{APT,non-stoi}$.

In order to have an objective quantitative comparison of the misfit stresses in the different samples, we examine the stresses along various paths in the different structures. To this end, we extract atoms enclosed by a cylinder of a certain radius and average the stress tensor of individual atoms. Figure 4 shows the stress along the face diagonal of a cross-sectional plane in different samples, for cylinder radii varying between 1 and 5 nm. This path has been carefully chosen, since it is the only path that is available, at least partly, in all samples. It is evident that the restrictive boundary conditions in samples *S*${}_{2Dp}$, *S*${}_{2DSEM}$, *S*${}_{APT,stoi}$ and *S*${}_{APT,non-stoi}$ lead to higher stresses than those observed in sample *S*${}_{cub}$. Nonetheless, the trend of the stress profile in samples *S*${}_{cub}$, *S*${}_{2Dp}$ and *S*${}_{2DSEM}$ is nearly the same. Furthermore, notwithstanding the identical boundary conditions in samples *S*${}_{APT,stoi}$ and *S*${}_{APT,non-stoi}$, significantly lower stresses can be observed in the non-stoichiometric sample *S*${}_{APT,non-stoi}$.

### 3.2. Evolution of a Dislocation Loop

The difference in the misfit stress distribution seen above, along with the BCs, must be expected to have a significant influence on the mechanical response of the different samples. To elucidate this, we investigate the evolution of a dislocation loop due to the internal stress state in the different structures. We point out that a tensile strain, whose magnitude is calculated from a theoretical value of the resolved shear stress to keep a dislocation loop stable (as defined by Equation (3)) and is a function of the loop radius, is applied whilst studying the evolution of the loop. This results in an additional tensile stress component in the x||[100] direction, which is equivalent in all samples, thereby allowing for a direct comparison of the simulations.

**Figure 5:**
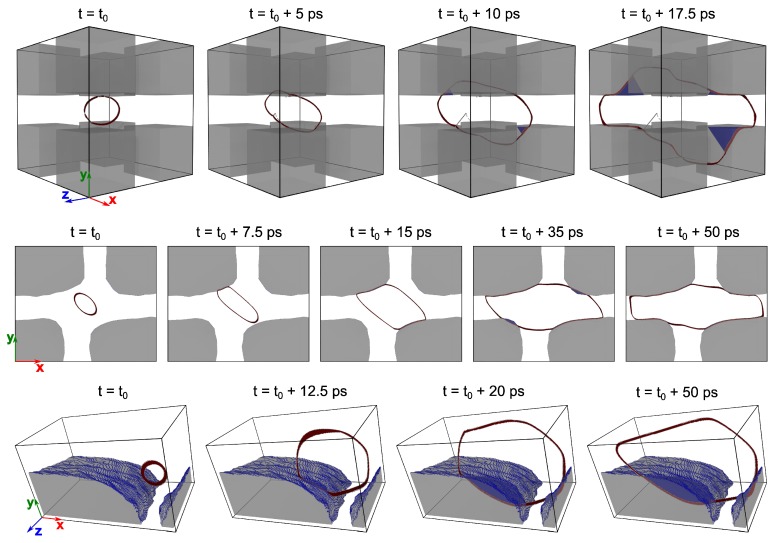
Evolution of a dislocation loop in the different atomistic samples. Top row: sample *S*${}_{cub}$ (Cube sample); Central row: SEM micrograph informed sample *S*${}_{2DSEM}$. Bottom row: APT-informed sample *S*${}_{APT,stoi}$ with the stoichiometric chemical composition. The $\gamma^{\prime }$ particle is enclosed by a semi-transparent surface. For clarity, only atoms identified as defects are shown. In samples *S*${}_{cub}$ and *S*${}_{APT,stoi}$, with fully-3D boundary conditions, the dislocation loop cuts through the $\gamma^{\prime }$ precipitate. By contrast, in sample *S*${}_{2DSEM}$ (with quasi-2D BC), only deposition of the loop is observed. Evolution of the loop in sample *S*${}_{2Dp}$ shows the same characteristics as that of sample *S*${}_{2DSEM}$ and is hence not shown here for the sake of brevity. Likewise, since the loop collapses even under applied strain in sample *S*${}_{APT,non-stoi}$ (APT-informed non-stoichiometric sample), the evolution of the loop is also not shown. The color code denotes defects as identified by AtomViewer [43,44]: red, stacking fault (lighter shade used to denote complex stacking fault in $\gamma^{\prime }$ phase); blue, antiphase boundary; white, other defects.

Snapshots of the MD simulations are presented in Figure 5. For clarity, the sample *S*${}_{cub}$ with PBCs is shifted so as to have the loop located roughly at the center of the structure. It is clearly visible in Figure 5 that for samples *S*${}_{cub}$, *S*${}_{2DSEM}$ and *S*${}_{APT,stoi}$, the internal stress state along with the applied strain leads to the expansion of the dislocation loop. In samples *S*${}_{cub}$ and *S*${}_{2DSEM}$, the expanding loop proceeds to deposit segments of a predominantly edge character on the $\gamma^{\prime }$ particle, which later cut through the interphase boundary to form a complex stacking fault (CSF). Furthermore, in sample *S*${}_{cub}$, these segments begin to cut through the particle at approximately 10 ps leaving behind an anti-phase boundary (APB) in their wake. With the passage of time, segments of a screw character also cut through the particle. The threading channel dislocation then reacts with its periodic image resulting in the annihilation of the corresponding segments (see Supplementary Movie S1 for more details). The superpartial dislocation in the $\gamma^{\prime }$ particle continues to cut further into the particle before reaching a stable position at roughly t=40 ps.

By contrast, in sample *S*${}_{2DSEM}$, the dislocation loop merely deposits itself (including segments of a screw character) on the $\gamma^{\prime }$ particles. The deposited segments then proceed to cut the IPB to form a CSF, but fail to cut through the particle to create any appreciable APB. Although a small APB was seen to form at roughly t=35 ps, the line tension of the threading dislocation in the channel pulls back the superpartial dislocation segment in the particle, thereby removing the APB. An almost identical evolution of the dislocation loop is seen in sample *S*${}_{2Dp}$ and is not presented here for the sake of brevity, but is presented as Supplementary Movie S2.

In the APT-informed sample with the stoichiometric chemical composition (*S*${}_{APT,stoi}$), only segments of a screw character can be deposited on the big particle shown in Figure 5. Similar mechanisms as those found in sample *S*${}_{cub}$ can be observed here. However, the higher stresses in the channels allow the deposited screw segments to cut through the particle forming the APB. Interestingly, an identical strain applied on the non-stoichiometric sample *S*${}_{APT,non-stoi}$ fails to stabilize the loop, causing it to collapse after a mere 5 ps.

The evolution of the dislocation loop in all samples can be found as Supplementary Material (as movies) accompanying the paper.

## 4. Discussion

It is well known that eigenstresses, i.e., residual stresses that remain in a material that is in global equilibrium with its surroundings, play an important role in determining the mechanical behavior of a structure [45]. Accurate prediction of the distribution of such eigenstresses is hence vital for reliable and robust simulation schemes (e.g., [46]), particularly multiscale frameworks, which link material microstructure at lower length scales with the mechanical properties at a continuum scale. In the current study, we investigate the distribution of eigenstresses as obtained from atomistic simulations of different samples with *γ*/$\gamma^{\prime }$ microstructures; the eigenstresses here being a direct consequence of the lattice misfit between the two phases. Furthermore, we perform a quantitative comparison of the atomistic stress state with that obtained from an FE simulation for an embedded cube microstructure (sample *S*${}_{cub}$). To the best of our knowledge, such a comparison has hitherto not been published in the literature for *γ*/$\gamma^{\prime }$ microstructures. We note that only the lattice misfit was used as a “fitting” parameter; no other assumptions are made for the stress distribution. Furthermore, the procedure to compute the stresses in the atomistic (energy minimization) and FE (thermal misfit) are quite different. The results show that the stress states of the atomistic and FE samples are almost identical. Such a consistent stress state, however, manifests itself only when PBCs are imposed on the structure in the FE model; simulations with periodic structures, albeit with homogeneous boundary conditions with special treatment to account for surface stresses [47], must hence be viewed critically.

Much can be argued about the computation of stresses in atomistic simulations, since the notion of an atomic stress, i.e., stress on an atom, remains rather controversial. Many formulations for atomic stresses have indeed been defined; see [48] for a recent review of the various formulations. Even with its inherent deficiencies, like, e.g., the presence of non-zero stresses normal to a free surface [49], the virial theorem is the most widely-used formulation and remains the standard against which other formulations are compared [50,51]. In the current work, we use the virial expression for the computation of atomic stresses in all of our samples. It is clear from the stress distributions in samples *S*${}_{cub}$ and *S*${}_{FE}$ that the virial expression suffices, at least for cases without free surfaces. For the samples *S*${}_{2DSEM}$, *S*${}_{2Dp}$, *S*${}_{APT,stoi}$ and *S*${}_{APT,non-stoi}$, however, the atoms that are fixed are removed from the calculation of the stress profile; hence, free surface effects are absent in the stress distribution.

The atomic stresses in the $\gamma^{\prime }$ particle are observed to vary strongly from atom to atom (see, e.g., Figure 3b). In general, there exists an inherent ambiguity in partitioning the total energy of the system to a so-called per-atom energy, from which an atomic stress can be directly derived. This is particularly true for the bi-atomic system of the $\gamma^{\prime }$ phase. Within the critical radius of 2 * $r_{cut}$, with $r_{cut}$ being the cutoff radius of the potential, all Al atoms sufficiently far away from an interface experience the same bonding environment. The same is, however, not true for Ni atoms; individual atomic planes in the (1 1 1) direction can be either Ni–Al or Ni only. Consequently, an apparent inhomogeneity is observed in the atomic stresses, which is particularly accentuated in sample *S*${}_{APT,non-stoi}$ due to non-stoichiometric chemical composition. These atomic stresses have little to do with their continuum counterpart and are averaged over a larger volume than that of individual atoms to obtain a smoother distribution. For the samples used in the current work, it was seen that an averaging volume of roughly 2 nm${}^{3}$ results in a smooth distribution of misfit stresses in the sample. Such spatial averaging is, however, sensitive to the placement of the averaging volume and can be circumvented by choosing appropriate kernel functions as weighting factors for the virial expression [48].

The internal stress values observed in the samples are seemingly high (>1 GPa). This is, however, a direct consequence of both the imposed strain, which itself is a function of the misfit between the two phases and the boundary conditions. The misfit of δ=1.4%, as defined by the interatomic potential used in the current work, is substantially larger than that (δ=0.2%) found in commercial Ni-base superalloys, which have significantly different alloying content than the binary Ni${}_{3}$Al $\gamma^{\prime }$ phase used in the current work. Furthermore, in reality, the two phases are not stacked one over the other as is done in the atomistic sample generation, but rather grown from a melt/solid solution, during which it is very likely that defects are created to reduce internal stresses.

The conformance of the stress state in the atomistic and FE samples *S*${}_{cub}$ and *S*${}_{FE}$ is indeed a very important result and has additional implications for atomistic simulations in general. The one-on-one correspondence of the stress state can be exploited to effectively expedite atomistic simulations, especially static relaxation, using, e.g., finite element informed atomistic simulations (FE2AT) [52], quasi-continuum (QC) [53] or similar methods ([54,55]), by imposing the displacement field obtained from the FE simulation onto the initial unrelaxed atomistic configuration.

It must be pointed out that technical Ni-base superalloys possess a negative misfit between the *γ*/$\gamma^{\prime }$ phases, resulting in compressive stresses in the *γ* channels. By contrast, we obtain tensile stresses in the *γ* channels, which is a direct consequence of the model material used in the current work, i.e., *γ*-phase modeled as fcc Ni and $\gamma^{\prime }$-phase modeled as L1${}_{2}$ Ni${}_{3}$Al. The stress state observed here is, nevertheless, of relevance to Co-base superalloys [56,57], which display a positive misfit. The procedure outlined in the current work to obtain the residual stress state, furthermore, remains the same.

The stress distribution in the different samples used in the current study are markedly different from one another, which furthermore, leads to differences in the evolution of a dislocation loop in the three samples. It can be argued that this difference in stress distribution is rather unsurprising, given that each sample is generated by a different processing route. However, certain general trends can be easily concluded from the results presented, by comparing like samples and delineating the effects of structural features and boundary conditions. For instance, the presence of curved interfaces relieves stress concentration (see Figure 3, samples *S*${}_{2DSEM}$ and *S*${}_{APT,stoi}$) that is usually observed along sharp corners (sample *S*${}_{2Dp}$) and also results in a smoother transition of stresses between the orthogonal and vertical channels. Furthermore, 2D/quasi-2D boundary conditions are ostensibly more restrictive than PBCs or fixed 3D boundary conditions. This is evident from the high stresses in the orthogonal channels of samples *S*${}_{2Dp}$ and *S*${}_{2DSEM}$. Additionally, in these samples, the dislocation loop fails to cut through the $\gamma^{\prime }$ particle and is merely deposited on the interface, since the line tension of the dislocation pulls it back and removes the minimal APB formed in the $\gamma^{\prime }$ phase.

The evolution of the dislocation loop is also influenced by image forces due to the boundary conditions imposed on the sample. Some general trends can be drawn from the results. Samples *S*${}_{cub}$ and *S*${}_{APT,stoi}$ with PBCs and fixed boundary conditions, respectively, result in image forces that are attractive and repulsive, respectively. In both cases, however, the dislocation loop cuts through the $\gamma^{\prime }$ phase. Nonetheless, we note that a conclusive statement on the influence of image forces on the evolution of the loop cannot be directly made, due to the difference in dimensions of the different samples.

The difference in stresses of samples *S*${}_{APT,stoi}$ and *S*${}_{APT,non-stoi}$ is, however, a direct consequence of variation in chemical composition. In the case of sample *S*${}_{APT,non-stoi}$, since the *γ* and $\gamma^{\prime }$ phases are no longer comprised entirely of Ni and Ni${}_{3}$Al, respectively, the local bonding environment of individual atoms changes drastically. This in turn changes the equilibrium lattice spacing and the elastic constants of the individual phases, significantly leading to lower stresses than those observed in sample *S*${}_{APT,stoi}$. As a result, the effective strain calculated for sample *S*${}_{APT,stoi}$ is insufficient to keep the loop from collapsing in sample *S*${}_{APT,non-stoi}$. A more detailed study on the influence of change in chemical composition on the observable cutting strength of $\gamma^{\prime }$ precipitates will be reported elsewhere.

A final note on BCs used in atomistic simulations: A robust generic simulation procedure requires, in addition to the numerical treatment of the underlying algorithm, primarily three main components: (i) a reliable model that accurately describes material behavior; (ii) a virtual sample that replicates structural and topological features found in real-world microstructures; and (iii) accurate BCs that reflect those experienced by real-world structures. The availability of an accurate interatomic potential (which, here, defines the material model) has long been recognized as a key element in atomistic simulations [58]. The incorporation of realistic microstructures, preferably a one-on-one reconstruction of those found in experiments, has also started to gain impetus over the past few years [21]. The imposition of accurate BCs, by contrast, is often neglected. In particular, quasi-2D BCs, which result from the usage of planar/columnar microstructures, are restrictive and result in material behavior that might not be observable in real-world scenarios. Such BCs have indeed been known to result in both false positives and negatives. For instance, deformation twinning was observed in nanocrystalline aluminum with columnar microstructures [59,60], but has been rarely observed in fully-3D microstructures [61]. In the current work, we see that such quasi-2D BCs result in a stress state that is not observed in samples with 3D (either PBC or fixed) BCs. The results of atomistic simulations with such quasi-2D BCs must hence be noted with caution.

We note in passing that the imposition of 2D/quasi-2D BCs may not be restrictive in the case of continuum-scale FE simulations, since the 3D nature of the problem may well be incorporated through the material model (e.g., [62,63]), as is indeed the case in multiscale frameworks, e.g., of crystal plasticity [64].

## 5. Conclusions

In this work, we have investigated the influence of idealized and experimentally-informed, more realistic, atomistic samples and accompanying BCs, on the internal stress distribution in *γ*/$\gamma^{\prime }$ microstructures, which results from the lattice misfit between the *γ*/$\gamma^{\prime }$ phases. Furthermore, we compare the idealized atomistic sample, *S*${}_{cub}$, with an equivalent FE sample, *S*${}_{FE}$, to ascertain the consistency of the resulting stress state.

The findings of the current work can be summarized as follows:

- The virial expression for the atomic stress results in a stress description that is consistent with FE simulations, even for bi-atomic systems like that found in idealized *γ*/$\gamma^{\prime }$ microstructures. This has been demonstrated by comparing the stresses in the embedded cube atomistic sample with those of an FE sample with PBCs and anisotropic elastic constants as defined by the interatomic potential.
- The near conformance of the stress state in the atomistic sample and the corresponding FE sample lets one conclude that FE simulations or concurrent multiscale models can be used to obtain the effective stress state in *γ*/$\gamma^{\prime }$ microstructures and, thus, expedite significantly expensive atomistic calculations.
- Significantly different stress states are observed in the *γ* and $\gamma^{\prime }$ phases of the different samples, which can essentially be ascribed to the sample generation procedure and the boundary conditions applied.
- Samples with PBCs lead to stresses that are quantitatively lower than samples with fully-3D fixed BCs. Qualitatively, however, the stress state, i.e., tensile stresses in channels parallel to the loading direction and compressive stresses in channels orthogonal to the loading direction, is, nevertheless, similar in the samples with PBCs or fully-fixed BCs.
- 2D/quasi-2D BCs can result in a fictitious stress state that may not be observable in full 3D setups and must be used with utmost care in atomistic simulations.
- The incorporation of experimental information into atomistic microstructures, e.g., topological features like curvature or variation in chemical composition, although necessary, may not be sufficient enough to provide improved insights into material behavior. The influence of accompanying BCs must also be accounted for.

## Figures and Tables

**Table 1:** Details on both the idealized and realistic *γ*/$\gamma^{\prime }$ samples used in the current work. The $\gamma^{\prime }$ volume fraction is computed using the number of atoms that constitute the $\gamma^{\prime }$ phase. The area fraction is computed in a similar fashion, but by using a thin slice of atoms in the (001) plane. Note that for the APT-informed samples, the area fraction (marked * in the table) depends on the place where this slice is taken from, and hence, a range is provided. The sample *S*${}_{FE}$ is the finite element sample, and all other samples are atomistic samples, which are grouped together for clarity. For a detailed description of the sample nomenclature, the reader is referred to the text in the article.

	Sample Name →	*S*${}_{FE}$	*S*${}_{cub}$	*S*${}_{2Dp}$	*S*${}_{2DSEM}$	*S*${}_{APT,stoi}$	*S*${}_{APT,non-stoi}$
Attributes ↓							
Sample dimensions (nm${}^{3}$)		100 × 100 × 100	100 × 100 × 100	150 ×125 × 50	150 × 125 × 50	75 × 45 × 44	75 × 45 × 44
Sample type		Idealized	Idealized	Idealized	Realistic	Realistic	Realistic
Number of atoms/elements		512,000	89,703,072	83,598,900	84,196,035	13,515,848	13,515,848
Boundary condition in *x*		PBC	PBC	*fixed*	*fixed*	*fixed*	*fixed*
Boundary condition in *y*		PBC	PBC	*fixed*	*fixed*	*fixed*	*fixed*
Boundary condition in *z*		PBC	PBC	PBC	PBC	*fixed*	*fixed*
Type of interfaces		Planar	Planar	Planar	Curved	Curved	Curved
Sharp edges/corners		present	present	present	absent	absent	absent
*γ*-channel size (nm)		25	25	25	≈25	≈15	≈15
$\gamma^{\prime }$ area fraction (along the (001) plane)		56.25%	56.25%	66.67%	62.3%	≈25.7%–≈46.8% *	≈25.7%–≈46.8% *
$\gamma^{\prime }$ volume fraction		42.2%	42.2%	66.67%	62.3%	34.6%	34.6%

## Supplementary Materials

The following are available online at www.mdpi.com/1996-1944/10/1/88/s1. Supplementary Material S1: Movie showing the evolution of the dislocation loop in sample *S*${}_{cub}$. Supplementary Material S2: Movie showing the evolution of the dislocation loop in sample *S*${}_{2DSEM}$. Supplementary Material S3: Movie showing the evolution of the dislocation loop in sample *S*${}_{2Dp}$. Supplementary Material S4: Movie showing the evolution of the dislocation loop in sample *S*${}_{APT,stoi}$. Supplementary Material S5: Movie showing the evolution of the dislocation loop in sample *S*${}_{APT,non-stoi}$.

## References

[1] Reed R.C. (2006). The Superalloys: Fundamentals and Applications.

[2] Van Sluytman J.S., Pollock T.M. (2012). Optimal precipitate shapes in nickel-base *γ*/*γ*^′^ alloys. Acta Mater..

[3] Murakumo T., Kobayashi T., Koizumi Y., Harada H. (2004). Creep behaviour of Ni-base single-crystal superalloys with various *γ*^′^ volume fraction. Acta Mater..

[4] Thorntona K., Akaiwab N., Voorhees P.W. (2004). Large-scale simulations of Ostwald ripening in elastically stressed solids: I. Development of microstructure. Acta Mater..

[5] Carroll L.J., Feng Q., Pollock T.M. (2008). Interfacial dislocation networks and creep in directional coarsened Ru-containing nickel-base single-crystal superalloys. Metall. Mater. Trans. A Phys. Metall. Mater. Sci..

[6] Kamaraj M. (2003). Rafting in single crystal nickel-base superalloys—An overview. Sadhana.

[7] Zhang J.X., Murakumo T., Koizumi Y., Kobayashi T., Harada H., Masaki S. (2002). Interfacial dislocation networks strengthening a fourth-generation single-crystal TMS-138 superalloy. Metall. Mater. Trans. A.

[8] Farkas D. (2013). Atomistic simulations of metallic microstructures. Curr. Opin. Solid State Mater. Sci..

[9] Bitzek E., Kermode J.R., Gumbsch P. (2015). Atomistic aspects of fracture. Int. J. Fract..

[10] Zhu Y., Li Z., Huang M. (2013). Atomistic modeling of the interaction between matrix dislocation and interfacial misfit dislocation networks in Ni-based single crystal superalloy. Comput. Mater. Sci..

[11] Zhu T., Wang C. (2005). Misfit dislocation networks in the *γ*/*γ*^′^ phase interface of a Ni-based single-crystal superalloy: Molecular dynamics simulations. Phys. Rev. B.

[12] Kohler C., Kizler P., Schmauder S. (2005). Atomistic simulation of the pinning of edge dislocations in Ni by Ni_3_Al precipitates. Mater. Sci. Eng. A.

[13] Zhu T., Wang C., Gan Y. (2010). Effect of Re in *γ* phase, *γ*^′^ phase and *γ*/*γ*^′^ interface of Ni-based single-crystal superalloys. Acta Mater..

[14] Djuansjah J.R., Yashiro K., Tomita Y. (2008). Computational study on misfit dislocation in Ni-based superalloys by quasicontinuum method. Mater. Trans..

[15] Xie H.X., Wang C.Y., Yu T. (2009). Motion of misfit dislocation in an Ni/Ni_3_Al interface: A molecular dynamics simulations study. Model. Simul. Mater. Sci. Eng..

[16] Ye X.J., Liu C.S., Zhong W., Du Y.W. (2015). Precipitate size dependence of Ni/Ni_3_Al interface energy. Phys. Lett. A.

[17] Prakash A., Guénolé J., Wang J., Müller J., Spiecker E., Mills M.J., Povstugar I., Choi P., Raabe D., Bitzek E. (2015). Atom probe informed simulations of dislocation-precipitate interactions reveal the importance of local interface curvature. Acta Mater..

[18] Dream3D. http://dream3d.bluequartz.net.

[19] NanoSCULPT. http://www.gmp.ww.uni-erlangen.de/nanoSCULPT.php.

[20] Moody M.P., Ceguerra A.V., Breen A.J., Cui X.Y., Gault B., Stephenson L.T., Marceau R.K.W., Powles R.C., Ringer S.P. (2014). Atomically resolved tomography to directly inform simulations for structure-property relationships. Nat. Commun..

[21] Dingreville R., Karnesky R.A., Puel G., Schmitt J.-H. (2016). Review of the synergies between computational modeling and experimental characterization of materials across length scales. J. Mater. Sci..

[22] Friak M., Tytko D., Holec D., Choi P.P., Eisenlohr P., Raabe D., Neugebauer J. (2015). Synergy of atom-probe structural data and quantum-mechanical calculations in a theory-guided design of extreme-stiffness superlattices containing metastable phases. New J. Phys..

[23] Wu W., Guo Y., Wang Y., Mueller R., Gross D. (2011). Molecular dynamics simulation of the structural evolution of misfit dislocation networks at *γ*/*γ*^′^ phase interfaces in Ni-based superalloys. Philos. Mag..

[24] Yashiro K., Naito M., Tomita Y. (2002). Molecular dynamics simulation of dislocation nucleation and motion at *γ*/*γ*^′^ interface in Ni-based superalloy. Int. J. Mech. Sci..

[25] Haghighat S.M.H., Eggeler G., Raabe D. (2013). Effect of climb on dislocation mechanisms and creep rates in *γ*^′^-strengthened Ni base superalloy single crystals: A discrete dislocation dynamics study. Acta Mater..

[26] Vattré A., Devincre B., Roos A. (2009). Dislocation dynamics simulations of precipitation hardening in Ni-based superalloys with high *γ*^′^ volume fraction. Intermetallics.

[27] Link T., Epishin A., Fedelich B. (2009). Inhomogeneity of misfit stresses in nickel-base superalloys: Effect on propagation of matrix dislocation loops. Philos. Mag..

[28] Meissonnier F.T., Busso E.P., O’Dowd N.P. (2001). Finite element implementation of a generalised non-local rate-dependent crystallographic formulation for finite strains. Int. J. Plast..

[29] Prakash A., Weygand S.M., Riedel H. (2009). Modeling the evolution of texture and grain shape in Mg alloy AZ31 using the crystal plasticity finite element method. Comput. Mater. Sci..

[30] Schmidt I. (2011). Numerical homogenisation of an elasto-plastic model-material with large elastic strains: Macroscopic yield surfaces and the eulerian normality rule. Comput. Mech..

[31] Heckl A., Neumeier S., Göken M., Singer R.F. (2011). The effect of Re and Ru on *γ*/*γ*^′^ microstructure, *γ*-solid solution strengthening and creep strength in nickel-base superalloys. Mater. Sci. Eng. A.

[32] Prakash A., Hummel M., Schmauder S., Bitzek E. (2016). NanoSCULPT: A methodology for generating realistic configurations for atomistic simulations. MethodsX.

[33] NanoSCULPT. https://bitbucket.org/arunpksh/nanosculpt/wiki/Home.

[34] Mishin Y. (2004). Atomistic modeling of the *γ* and *γ*^′^-phases of the Ni-Al system. Acta Mater..

[35] Bitzek E., Koskinen P., Gähler F., Moseler M., Gumbsch P. (2006). Structural relaxation made simple. Phys. Rev. Lett..

[36] Bitzek E., Brandl C., Weygand D., Derlet P.M., van Swygenhofen H. (2009). Atomistic simulation of a dislocation shear loop interacting with grain boundaries in nanocrystalline aluminium. Model. Simul. Mater. Sci. Eng..

[37] Beeler J.R. (1983). Radiation Effects Computer Experiments.

[38] Scattergood R.O., Bacon D.J. (1982). The strengthening effect of voids. Acta Metall..

[39] Glatzel U., Feller-Kniepmeier M. (1989). Calculations of internal stresses in the *γ*/*γ*^′^ microstructure of a nickel-base superalloy with high volume fraction of the *γ*-phase. Scr. Metall..

[40] Fedelich B. (1999). A microstructure based constitutive model for the mechanical behavior at high temperatures of nickel-base single crystal superalloys. Comput. Mater. Sci..

[41] Allen M.P., Tildesley D.J. (1996). Computer Simulation of Liquids.

[42] Stukowski A. (2010). Visualization and analysis of atomistic simulation data with OVITO—The Open Visualization Tool. Model. Simul. Mater. Sci. Eng..

[43] Begau C., Hartmaier A., George E.P., Pharr G.M. (2011). Atomistic processes of dislocation generation and plastic deformation during nanoindentation. Acta Mater..

[44] Amodeo J., Begau C., Bitzek E. (2014). Atomistic simulations of compression tests on Ni_3_Al nanocubes. Mater. Res. Lett..

[45] Withers P.J., Bhadeshia H.K.D.H. (2001). Residual stress. Part 2—Nature and origins. Mater. Sci. Technol..

[46] Korsunsky A.M., Guénolé J., Salvati E., Sui T., Mousavi M., Prakash A., Bitzek E. (2016). Quantifying eigenstrain distributions induced by focused ion beam damage in silicon. Mater. Lett..

[47] Preußner J., Rudnik Y., Brehm H., Völkl R., Glatzel U. (2009). A dislocation density based material model to simulate the anisotropic creep behavior of single-phase and two-phase single crystals. Int. J. Plast..

[48] Admal N.C., Tadmor E.B. (2010). A unified interpretation of stress in molecular systems. J. Elast..

[49] Cheung K.S., Yip S. (1991). Atomic-level stress in an inhomogeneous system. J. Appl. Phys..

[50] Zimmerman J.A., Jones R.E., Klein P.A., Bammann D.J., Webb E.B., Hoyt J.J. (2002). Continuum Definitions for Stress in Atomistic Simulations.

[51] Zimmerman J.A., Webb E.B., Hoyt J.J., Jones R.E., Klein P.A., Bammann D.J. (2004). Calculation of stress in atomistic simulation. Model. Simul. Mater. Sci. Eng..

[52] Möller J.J., Prakash A., Bitzek E. (2013). FE2AT: Finite element informed atomistic simulations. Model. Simul. Mater. Sci. Eng..

[53] Miller R.E., Tadmor E.B. (2002). The Quasicontinuum Method: Overview, applications and current. J. Comput.-Aided Mater. Des..

[54] Curtin W.A., Miller R.E. (2003). Atomistic/continuum coupling in computational materials science. Model. Simul. Mater. Sci. Eng..

[55] Miller R.E., Tadmor E.B. (2009). A unified framework and performance benchmark of fourteen multiscale atomistic/continuum coupling. Model. Simul. Mater. Sci. Eng..

[56] Sato J., Omori T., Oikawa K., Ohnuma I. (2006). Cobalt-base high-temperature alloys. Science.

[57] Bauer A., Neumeier S., Pyczak F., Göken M. (2010). Microstructure and creep strength of different *γ*/*γ*^′^-strengthened Co-base superalloy variants. Scr. Mater..

[58] Srolovitz D., Vitek V. (1989). Atomistic Simulation of Materials: Beyond Pair Potentials.

[59] Yamakov V., Wolf D., Phillpot S., Mukherjee A., Gleiter H. (2002). Dislocation processes in the deformation of nanocrystalline aluminium by molecular-dynamics simulation. Nat. Mater..

[60] Yamakov V., Wolf D., Phillpot S.R., Gleiter H. (2002). Deformation twinning in nanocrystalline Al by molecular dynamics simulation. Acta Mater..

[61] Frøseth A.G., Derlet P.M., van Swygenhoven H. (2005). Twinning in nanocrystalline fcc metals. Adv. Eng. Mater..

[62] Prakash A., Nöhring W.G., Lebensohn R.A., Höppel H.W., Bitzek E. (2010). A multiscale simulation framework of the accumulative roll bonding process accounting for texture evolution. Mater. Sci. Eng. A.

[63] Segurado J., Lebensohn R., Lorca J.L., Tomé C.N. (2012). Multiscale modeling of plasticity based on embedding the viscoplastic self-consistent formulation in implicit finite elements. Int. J. Plast..

[64] Roters F., Eisenlohr P., Hantcherli L., Tjahjanto D.D., Bieler T.R., Raabe D. (2010). Overview of constitutive laws, kinematics, homogenization and multiscale methods in crystal plasticity finite-element modeling: Theory, experiments, applications. Acta Mater..

